# *Flow*: Statistics, visualization and informatics for flow cytometry

**DOI:** 10.1186/1751-0473-3-10

**Published:** 2008-06-17

**Authors:** Jacob Frelinger, Thomas B Kepler, Cliburn Chan

**Affiliations:** 1 Computational Biology and Bioinformatics Program, Duke University, Durham, NC 27705, USA; 2 Center for Computational Immunology, Department of Biostatistics & Bioinformatics, Duke University Medical Center, 2424 Erwin Road, Hock Plaza Suite G06, Durham, NC 27705, USA; 3 Department of Immunology, and Department of Statistical Science, Duke University, Durham, NC 27705, USA

## Abstract

*Flow *is an open source software application for clinical and experimental researchers to perform exploratory data analysis, clustering and annotation of flow cytometric data. *Flow *is an extensible system that offers the ease of use commonly found in commercial flow cytometry software packages and the statistical power of academic packages like the R BioConductor project.

## Goals

### Users

Provide a platform for clinical and laboratory researchers to perform exploratory data analysis, clustering and annotation of flow cytometric data.

### Developers

Provide a plugin framework and API so that the software can be easily extended and customized.

## Background

Flow cytometry is a high throughput method extensively used in both experimental and clinical settings for characterizing cell phenotypes, especially in the disciplines of hematology, immunology and infectious diseases. In flow cytometry, intracellular or cell surface molecules (markers) in a cell population are tagged with one or more fluorescent dyes, then streamed down a flow cell where the fluorochromes are activated by multiple lasers [1]. Since each fluorochrome emits light of a specific color when activated, the density of tagged molecules on each cell can be estimated. With recent technological advances, a single session may collect high-resolution digital events from up to a million cells, each labeled with up to 20 different fluorochromes, resulting in a data set that is both high volume and multi-dimensional [2]. Polychromatic flow cytometry (PFC) is the only currently available assay that can track multiple functional responses simultaneously, on both single cell and population levels [3], and uses include immunophenotyping, monitoring of complex immune responses in HIV patients and vaccine trials and characterization of cell signaling with labeled antibodies to phosphoproteins. With today's software, flow cytometric datasets are typically analyzed by manually circumscribing (gating) interesting regions using a sequence of 1 or 2-dimensional plots to identify different subsets. The main problem with manual gating is that it is highly operator-dependent and subjective, with the choice of gating sequence and position chosen by expertise, making replication of results by a different laboratory extremely difficult. The lack of software suitable for experimentalists and clinicians to efficiently and robustly characterize flow data is a bottleneck in the use of PFC. Since the reliable identification of cell subsets is necessary for further investigation of their function, this problem affects downstream research as well.

The analysis of PFC is largely a process of trying to find structure in a high-dimensional space, and visualization is an invaluable aid to such exploratory data analysis. In addition to the standard histogram and dot plot views of flow data, *Flow *also provides graphics to visualize data in three dimensions, along with the ability to rotate, pan and zoom into or out of the 3D view. The motivating idea is that if a subset of cells forms a distinct visual subgroup that can be demarcated visually with sequential gating, it should also be possible in principle to extract the subset with the appropriate statistical model. In practice, accurate gating is often only possible with extensive expert knowledge and by comparison with both negative and positive controls, and formidable statistical challenges exist. Nevertheless, we believe that statistical modeling can ameliorate much of the tedium and subjectivity inherent in gating on PFC data, and that *Flow *is a useful medium to increase awareness of such methods among the flow community.

*Flow *is an open source software application for flow cytometric analysis that integrates tools for exploratory data analysis, clustering and annotation of flow cytometric data sets. Together, these provide powerful new tools for the experimentalist or clinician to analyze flow cytometric data in a different way – one that will complement and perhaps supersede the traditional strategy of sequential gating. While the current features of *Flow *are naturally biased toward our own research interests, we have implemented features that allow the software to be used for traditional analysis by gating. As *Flow *is both open source and extensible by design with third-party plugins, we encourage the user community to provide additional functionality, and provide a developer's guide to the API on our website [4].

There has been increasing interest in the potential utility of statistical clustering and specifically the use of mixture models for flow cytometry, as shown by recent publications in Cytometry A, the official journal of the International Society for Analytical Cytology (ISAC) [5-7]. Flow is a software application and interface to expose research and clinical cytometrists to these powerful techniques that will surely become more important as the complexity and dimensionality of flow technology increases.

## Design

The overall design for Flow is based on the Model-View-Controller (MVC) design pattern [8] that separates the visual display and controls from the data model cleanly. The core of the Model is implemented using HDF5 [9], which provides a well-documented and flexible API for hierarchical data structures, augmented with functions to process the data through various statistical procedures. The Views and Controllers are implemented by hooking up a portable graphical user interface toolkit to the Model using the event-driven model provided by all such toolkits.

Flow is developed primarily in Python, a dynamic object-oriented programming language. Python was chosen because it is open-source and has modules to handle common tasks, as well as excellent numerical and scientific support with the numpy and scipy modules [10]. Choosing a high level language reduced development time and made it easy to port the application to multiple operating systems.

Core routines in the base system handle data management, while plugins for IO, statistics and visualization provide the remaining functionality. This decoupling facilitates independent development of new functionality, and encourages an evolutionary approach to software development. The extensibility of Python allows such plugins to be developed in a variety of languages. For example, several of the current plugins take advantage of this by using C/C++ for computationally intensive routines and R for specific statistical algorithms. As a result, developers can easily implement new functionality to fit their specific needs. However, the demarcation between plugins and base system is mainly relevant for *developers*; plugins are integrated into the system in such a way as to be essentially transparent to the *user*.

## Implementation

The GUI was developed using wxPython [11] a wrapper for the wxWidgets cross-platform toolkit [12]. wxWidgets is a modern event-driven windowing toolkit with a native look and feel on different operating systems. It provides the menus, toolbars and other windowing widgets that are responsive to keyboard or mouse events to provide the functionality users of modern software expect. Importantly, wxPython is thread-aware, allowing us to write long running processes as threads, preventing the UI "lock-up" that would otherwise occur.

The main functionality provided by the base system is GUI widgets that provide links to plugins. A key widget is the Control frame, which represents the entire flow cytometry analysis session as a tree widget and shows the entire Model in our MVC design. The Control frame offers a powerful and intuitive interface for complex multi-stage analysis of flow cytometry data, since it displays an editable and selectable view of the entire process history of the currently selected data set. In addition to linear processing, different analysis pipelines can be performed on the same FCS data, and the resulting outcome compared using the Control frame.

The Control Frame also provides a simple and intuitive interface for sample annotation, allowing the user to maintain a record of experimental metadata. Any non-terminal node in the control tree is a group that can be annotated by arbitrary key-value pairs. Selecting the Annotate item in the contextual (right click) menu will present an editable table of current annotations (i.e. key value pairs) for the selected node.

To reduce storage requirements, the children of a node inherit data, annotations and cluster labels associated with the parent node unless over-ridden in the child group. However, other attributes are not inherited, as this may not always make sense. Instead, we have provided a facility to copy and paste attributes from one group to another by using the right click menu.

In addition, certain functionality that involves direct manipulation of the data and is almost invariably necessary for flow cytometric analysis is part of the base system. In particular, data compensation, sub-sampling and transformations are implemented as part of the base system. Other functionality such as input and output (IO), statistics and visualization graphics are implemented as plugins.

To explore high dimensional flow cytometric datasets, several alternative analytical routes may be chosen, for example, k-means clustering or mixture modeling. Each of these routes may have common pre-processing requirements and involve several steps that must be taken in the correct sequence. As a consequence, the processing tasks performed during an analysis session has a tree topology, and an appropriate data structure is required to store the session data and metadata for archiving or further analysis at a later date. We therefore chose the HDF5 data format to implement the storage of an analysis session. HDF5 is a mature library and hierarchical file format for storing scientific data developed by the NCSA. Since the HDF5 format is intrinsically hierarchical, library implementations for all the necessary operations to navigate, manipulate and search a tree data structure are already provided. HDF5 also supports metadata annotation and efficient storage of large data matrices. Conveniently, we can manipulate the HDF5 structure using PyTables [13], a Python wrapper for the HDF5 library routines. This also allows our data to be created and/or analyzed by a variety of tools that support the HDF5 file format.

### Data input and pre-processing

*Flow *has plugins that allow the reading of FCS 2.0, FCS 3.0, CSV and HDF5 file formats. A new HDF5 structure is created for all other file formats and the relevant data and metadata copied over. Results of data transforms and statistical analysis are appended to the HDF5 structure that is saved on request or upon exit from the program.

Because of spectral overlaps between different fluorochromes in the detection channels, it is necessary to compensate for spillover between channels, using a set of control samples stained with single fluorochromes. The Compensate menu option has a single "Apply Compensation" item. If there is a pre-existing compensation matrix in the FCS or HDF5 file, it will use that as the initial matrix displayed. Otherwise, an identity matrix with the correct number of dimensions will be created. The matrix shown can be edited, and changes to the compensation matrix dynamically update an associated dot plot to provide feedback on the effects of the modification.

Sub-sampling reduces the dimensionality of the data by removing columns or rows that are not of interest. Sub-sampling is generally used to create a smaller data set for pilot statistical studies in which speediness is traded off for some loss in accuracy. The Sample menu provides a simple interface to select columns of interest using a checklist box. Sub-sampling of rows can be done by explicit specification of rows using a [start: stop: stride] notation, or random sampling (with or without replacement) of a fixed number of events.

Transformations modify the actual values of events by applying some mathematical operation to the data. Unlike sub-sampling, transforms do not change the number of elements in the data set. Transformations implemented in the prototype include the general scale, normal scale, linear, quadratic, clip, log and log10 transforms, as well as the arcsinh, biexponential, hyperlog [14] and logicle [15] transforms that are specialized for flow cytometry data. In particular, linear FCS data typically needs to be transformed using the hyperlog or logicle algorithms prior to visualization and model fitting. All transforms are implemented using just the python numerical libraries numpy and scipy.

Projections take the data from the original *n*-dimensional *source *space into a *k*-dimensional *target *space, in which the target axes are typically linear combinations of the source axes. The Projections menu currently includes two of the widely used popular projections for reduction of dimension – principal components analysis (PCA) and independent components analysis (ICA). PCA transforms the data to a new coordinate system such that the greatest variance by any projection of the data comes to lie on the first coordinate (called the first principal component), the second greatest variance on the second coordinate, and so on. ICA separates a multivariate signal into additive sub-components by maximizing the statistical independence of the estimated components. These projections were implemented by simply wrapping the R library *fastICA *[16] functionality using *Rpy *[17], a great example of how Python's glue characteristic can dramatically reduce development time. Alternative versions of the PCA and ICA implemented using the Python module MDP (Modular toolkit for Data Processing) [18] are also provided and appear in the Projections menu as PcaPy and IcaPy.

### Exploratory data analysis

In addition to standard summary statistics that calculate the min, max, mean, median and standard deviations of each column, 2 and 3-D graphics plugins are provided for exploratory data analysis.

The 2D visualization plugins are developed using the Matplotlib toolkit [19], which provides object-oriented scientific plotting capabilities. 3D graphics are developed using the python wrappers for the Visualization Toolkit (VTK) [20] that is basically a high level interface to OpenGL routines designed around scientific visualization. Both toolkits are object-oriented and integrate well with wxPython, allowing the development of responsive displays necessary for gating.

Basic graphics developed with these toolkits and provided by *Flow *are the histogram, dot plot and 3D spin plot for visualizing the density of the currently selected group in the Control Frame. The histogram graphic provides an overlay option for comparing distributions from comparable channels of two different data sets. The dot plot is a 2D density plot, and this has options to overlay density contours, as well as confidence ellipses for data fitted using statistical mixture models. By default, the dot plot presents data using a density-dependant heat map, but this is over-ridden if cluster labels are available, in which case unique colors are assigned to events coming from specific clusters. A simple polygonal gating mechanism has been implemented to capture an irregularly shaped region. Alternatively, gating by quadrant is also an option, and the percentages of events in each quadrant are shown in the graph title. The 3D density or spin plot may be unfamiliar to most experimentalists, but is a simple extension of the dot plot into 3 dimensions. To help visualize the underlying structure of the data better, the spin plot can be rotated, translated and zoomed by left, middle and right click dragging the mouse respectively. A resizable box can be used for gating in 3D just as in 2D. Similar to the 2D density plot, events from each cluster are assigned unique colors if a labeling exists in the selected group. Finally, both the 2D and 3D plots allows individual or groups of components classified statistically to be extracted, using either the component label or assigned color for selection ("statistical gating"). All the variants of the density plots have a consistent design – the markers/projections to be plotted are selected with radio boxes, and visual gating is available from the Gating menu. Plotted graphics can be exported as Portable Network Graphics (PNG), Postscript (PS) or Portable Data Format (PDF) files, suitable for inclusion in a manuscript

Additional visualization options can be added as plugins – for example, an implementation of box-whiskers plots to summarize the location and scale characteristics of data in each channel is also provided as a separate plugin. High-dimensional data visualization is a well-developed field, and researchers interested in more sophisticated features may consider implementing a plugin to interface the GGobi [21] library that provides cutting edge visualization tools.

### Clustering

Our main research focus is on the use of statistical mixture models to cluster and classify flow cytometric data automatically. One of the simplest algorithms for clustering is the *k-*means algorithm, which we implemented trivially using the *kcluster *routine from the PyCluster [22,23] module (also available from BioPython [24] We have also implemented a kernel density estimation (KDE) plugin that uses the mean-shift algorithm to find the modes of the multivariate density, and subsequently assigns events to a mode using a simple nearest neighbor heuristic. Slightly more sophisticated is the use of mixture models to model the density, and we have also provided a plugin that performs Markov chain Monte Carlo (MCMC) sampling for density estimation.

The KDE and MCMC routines are provided by a shared library that is interpreted by Python as a module, generated by writing custom C++ code and wrapping with the Boost Python [25] libraries. Parameters for these routines can be entered via a dialog box, with sensible default values provided as far as possible. All the clustering routines result in labeling of the data – each event is assigned a number depending on which component it is most closely associated with. These labels are stored in the associated HDF5 file and provide a link between the density estimation and both the visualization and data management components. In addition, the routines may also generate new data that is stored in the HDF5 file, for example, MCMC fitting results in the creation of a new child data group with entries for the mixture proportions π, the mixture means μ and the mixture covariance matrices Σ.

### Ontology integration

One of the main problems with flow cytometry today is the lack of standardization, both in the analysis process as well as in reporting the outcome of such analysis. By using formal statistical procedures such as density estimation, we hope to reduce the element of subjectivity in identifying cell subsets. Even so, different laboratories may use different names for very similar cell phenotypes, particularly if the phenotype represents one of many different possible activation states or differentiation end points.

We propose to use a well-defined common vocabulary that describes the objects and relations of interest formally, so as to minimize the potential for confusion. Ontologies aim to provide such common vocabularies, and we believe that *Flow *is unique among flow cytometric packages in integrating the Cell Ontology [26] of the Open Biomedical Ontologies (OBO) group [27] to standardize the labeling of cell subsets.

The results of statistical analysis can typically be described as partitioning the flow cytometric events into clusters. For example, the kmeans algorithm naturally gives rise to k clusters, while the concept of *modal *clusters can be used for both kernel density and mixture model approaches. Recall that the various density estimation algorithms all partition the dataset into a much smaller subset of event clusters. Initially, these clusters are simply assigned numerical labels. We have integrated the Cell Ontology into the application so that right clicking on a label and selecting "Edit label" brings up a dialog showing the Cell Ontology hierarchy from which an appropriate cell type can be chosen. Of course, an arbitrary name can also be given if none of the Cell Ontology classes are suitable. Since the Cell Ontology cell types are unique and well defined, labels assigned using the ontology will not be ambiguous.

In addition to providing a common peer-reviewed vocabulary, the use of ontologies significantly expands the scope for automated processing and machine learning in flow cytometry. Such assigned cell type labels together with their statistical characterization can be saved to a relational database, forming a valuable training set for the classification of future flow cytometric data.

In practice however, the current repertoire of Cell Ontology is probably not sufficiently detailed for most flow cytometric annotations, and users may have to augment the OBO file with their own cell phenotype classes, which is easily done with either a text editor or the DAGEdit ontology editor provided by the OBO Foundation. While this obviously detracts from the purpose of having a *common *vocabulary, such augmented or custom ontologies can be easily shared and additional cell phenotypes proposed for inclusion in the Cell Ontology, thus benefiting both the flow cytometric community as well as the Cell Ontology.

### Grouping and batch operations

In many situations, typically the same gating or transformation needs to be applied to multiple data sets. To facilitate this, *Flow *supports batch operations that repeat the last performed gating or transformation data on a selected set of groups. This is accessible from the "Batch" option from the right click menu, which in turn opens a dialog box for the selection of groups to batch process on.

## Workflow

### Use case

**Figure 1:**
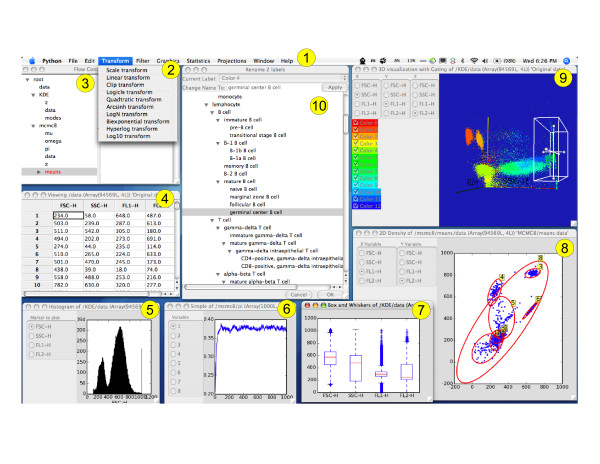
Screenshot of OS X version of *Flow *showing major components: 1) Menu bar, 2) Transformation menu, 3) Control window, 4) Table of data values, 5) Histogram, 6) Trace plot showing MCMC convergence, 7) Box and whiskers plot, 8) 2D density plot with confidence intervals for Bayesian mixture of Gaussians, 9) 3D density plot with box gate and 10) integrated Cell Ontology for cell subset labeling.

*Flow *is designed to integrate ideas from the fields of exploratory data analysis, clustering and annotation into a user-friendly platform for sophisticated analysis of PFC data. In this section, we walk through the analysis of a data set, and show how the various features of Flow are used together to create a powerful tool for biologists and clinicians. A screenshot of the software is provided in Figure 1 showing the major components described in this walk-through.

Starting up the program results in display of the Control Window, which shows the underlying data processing model as a tree in the left panel, with details and metadata for the currently selected node in the tree in the right panel. Each selected node can be edited, copied, cut, deleted or renamed using either the Tree or context ("right click") menus. On startup, only the root node is displayed in the left panel, and the right panel is blank. Each subsequent data operation will typically result in the creation of a new child group, which can also be selected, inspected, or manipulated using the Tree or context menus.

**Figure 2:**
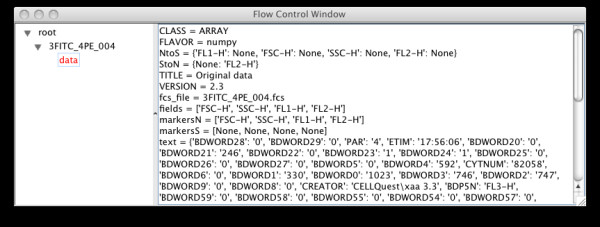
Control window after loading in an FCS file showing associated metadata automatically extracted from the FCS file.

**Figure 3:**
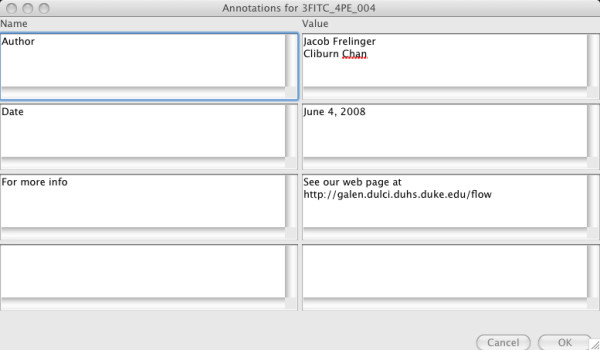
Adding annotations to a group.

Analysis of a data set typically begins with the loading of a data set exported from a flow cytometer in the FCS 2.0 or FCS 3.0 binary data format [28]. This is done by choosing the menu option File|Load FCS data, or Load all FCS files in directory for batch uploading. This will result in each FCS dataset appearing as a composite node or group with the actual data as an atomic component or leaf. Metadata about the dataset gleaned from the FCS file is stored as attributes of the data, and can be seen by selecting the data node (Figure 2). The group can also be annotated with metadata (Figure 3).

**Figure 4:**
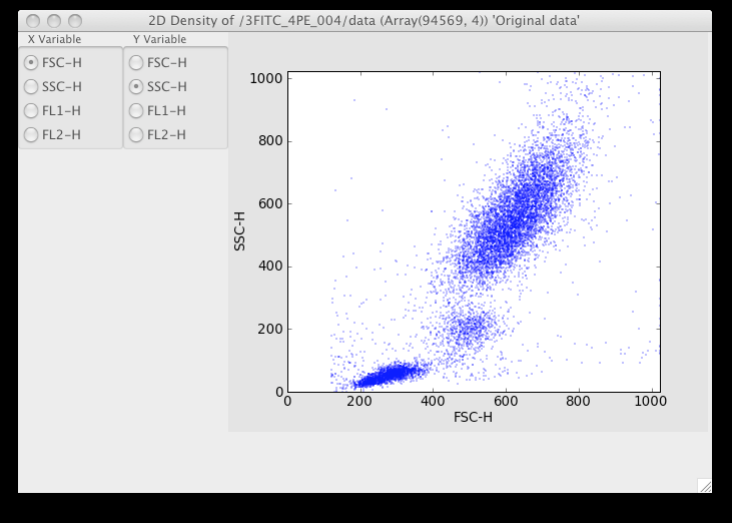
2D graphics showing FSC/SSC projection. Clicking on the radio buttons changes the projected dimension.

If necessary, the data can now be compensated using the Compensation menu, and then transformed appropriately using the Transformation menu. Selecting particular columns or rows using the Sample menu will reduce the size of the data set. For exploratory data analysis, it is now useful to select one or more options from the Graphics menu to visualize the data in 1, 2 or 3-D projections (Figure 4). Alternatively, the independent or principal components projections can be visualized by first selecting an option from the Projection menu.

**Figure 5:**
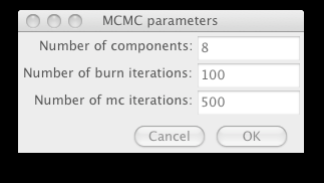
The options dialog for data clustering using a statistical mixture model and MCMC that is displayed on choosing the Statistics|Bayes option from the menu.

**Figure 6:**
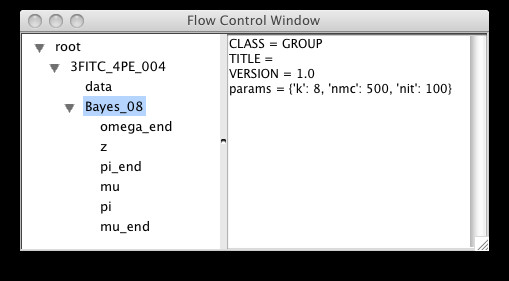
Results from the MCMC are automatically appended to the current analysis tree, along with metadata about the parameters chosen.

**Figure 7:**
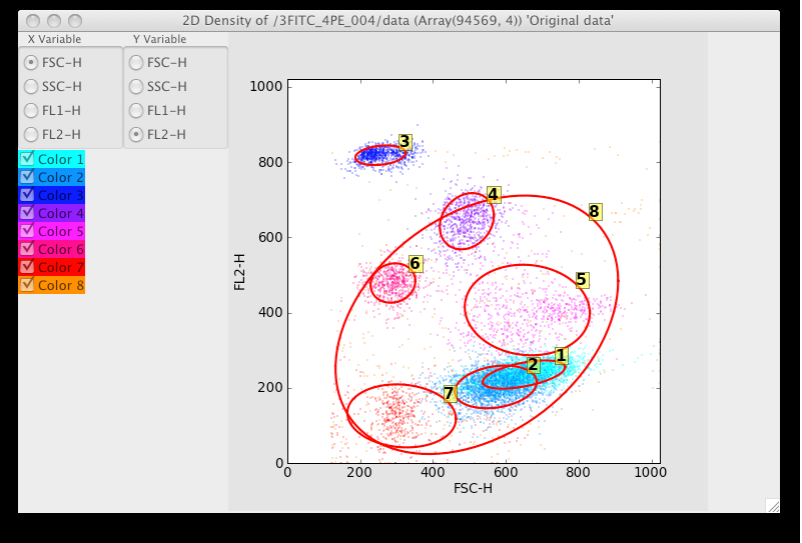
2D projection when the Bayes_08 data set is selected in the Control Panel. Confidence ellipses have also been added by choosing the Visuals|Confidence Ellipse menu option.

To cluster the data, choose the k-means, KDE or Bayes (statistical mixture modeling using MCMC) options from the Statistics options. The KDE and Bayes options can take a while to complete, and hence are implemented as threaded operations – they return control to the user while processing continues in the background. In particular, MCMC is highly computationally intensive, and it is often necessary to sub-sample the data using the Sample menu options to complete processing in a reasonable time for large datasets. The end result of all the statistical operations is a labeling of each data row vector ("point") with its assigned cluster, together with other generated summary data specific for each option (for example, the Bayes option will result in a covariance matrix for each cluster). See Figures 5, 6 and 7 for screenshots illustrating the use of the Bayes option.

**Figure 8:**
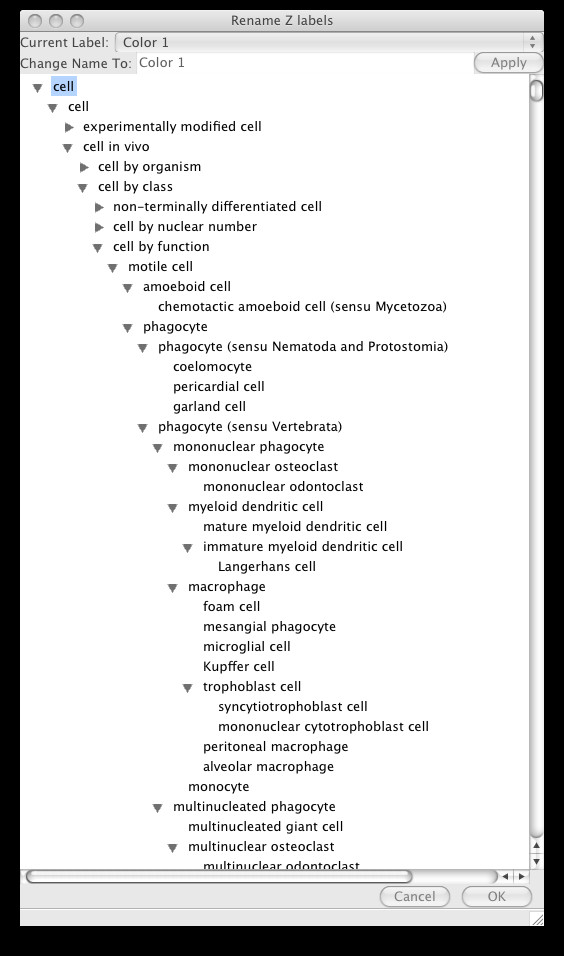
The Cell Ontology labeling menu that is displayed on right-clicking and selecting "Edit label" on any label in the 2D or 3D plots.

**Figure 9:**
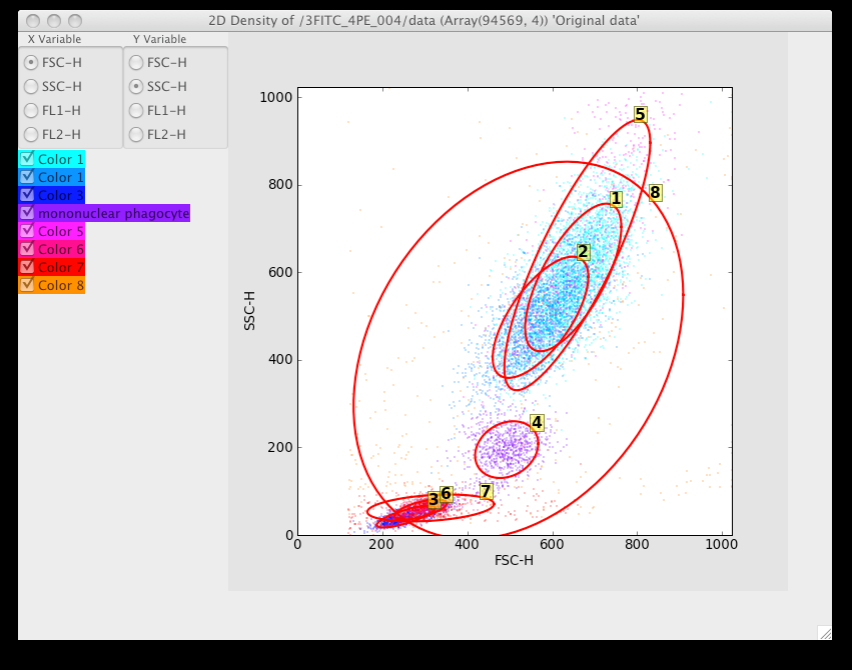
Component 4 has been relabeled as Mononuclear Phagocyte using the Cell Ontology menu.

After labeling, the 2D and 3D density graphics will use the label information to color each cluster of data points with a unique color. A simple form of statistical gating can now be performed by checking or un-checking the desired components in the left panel of the graphics window. If a component can be identified with a biological cell subset, the assigned label can be replaced with a more informative one by right clicking on the label and choosing Edit Z labels. This brings up a window with the cell types available in the Cell Ontology as an is-a hierarchy, from which the appropriate biological classification can be selected. See Figures 8 and 9 for an example of relabeling using the Cell Ontology.

**Figure 10:**
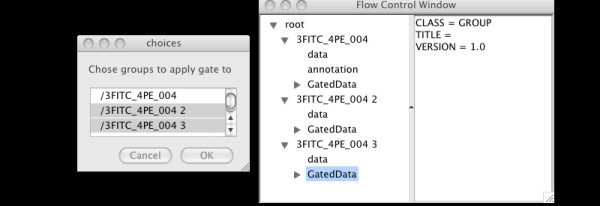
Batch gating on multiple data samples showing group selection dialog.

Finally, we illustrate a simple example of batch operations. First we open three FCS files, then draw a quadrant gate to extract a subset of the events from group 1. Now selecting the "Batch" option in the right click menu will bring up a dialog from which we can select groups 2 and 3, and clicking "OK" will result in the same gate being applied to Groups 2 and 3. See Figure 10 for this.

Upon completion of data analysis, the entire session can be saved as an HDF5 file by selecting File|Save HDF file. The program will also prompt to save the session before quitting if there are unsaved changes.

## Discussion and conclusion

Current flow cytometric software falls into two classes with essentially disjoint sets of users – traditional (mostly commercial) software (e.g. FlowJo [29], FCSExpress [30], FACSDiva [31]) that are easy to use but offer few statistical and visualization facilities beyond summary statistics and variants of the histogram and dot plots; and statistical software (e.g. rflowcyt and flowCore packages of the R BioConductor project [32]) that offer advanced statistical and visualization capabilities but require programming and statistical expertise.

There has recently been much interest in the potential application of automated multivariate statistical algorithms to flow cytometry [5-7], and we believe that *Flow *would provide a useful interface for these techniques, and encourage the dissemination and adoption of advanced statistical processing. We have developed the *Flow *software to meet the needs for exploratory data analysis and statistical modeling in PFC, in a package sufficiently intuitive for non-statisticians to use productively, so that it can be used by the experimentalists and clinicians who perform flow cytometric analysis routinely in their work. In addition, the emphasis on statistical mixture modeling for density estimation and ontological integration is, as far as we are aware, unique to *Flow*.

While *Flow *does not require any programming skills or detailed understanding of statistical methodology to use, it does require a conceptual understanding of the various statistical options to use effectively. We believe that these concepts can be taught to flow cytometrists, and suggest that software like *Flow *would form a useful adjunct for building the appropriate statistical intuition. A safeguard provided by *Flow *is that the entire history of the analysis can be recorded and transparently viewed in the Control Frame, allowing auditing of the analysis performed.

Another possible issue with *Flow *for naive users is the potentially large number of options available, and each menu option may in turn require specification of various parameters, albeit often with sensible default values provided. In contrast, commercial software is often developed mainly for ease of use – for example, a default transformation of linear data may automatically be applied which is invisible to the user and outside of the user's control. We believe that making the choices explicit where a statistical decision is necessary results in more informed analysis, but this unfortunately does result in a less streamlined procedure for analysis. We are currently exploring the option of allowing the user to record macros that would combine explicit choice with a streamlined analysis procedure.

One limitation of Flow is that the analysis is stored in an HDF5 format, and hence not readable by traditional flow cytometry software. We did not consider this benefit to be worth the trouble of developing an appropriate interface for storing and acquiring analytical results in the binary FCS format. An attractive solution would be to write a routine that translates the HDF5 data into the GatingML markup language [33], which provides an standard well-documented format for flow data exchange that is likely to be supported by other flow cytometry software in the near future. Users are encouraged to develop such plugins for their own needs and contribute to the further development of the software.

*Flow *is a software platform for the exploration, analysis and curation of PFC data that treads a middle ground between user-friendliness and power. The overall architecture is object-oriented and modular with most functionality provided as plug-ins. The code is mostly written in Python to enable rapid development, but computationally intensive numerical routines can be written in a compiled language like C++ or Fortran and provided as plugins. *Flow *runs on multiple platforms to reach a broad audience, and we expect that its open source release will encourage its rapid development via user-contributed plugins, and hence become ever more useful to the flow cytometry community.

## Availability and requirements

Project name: Flow

Project home page: 

Operating system: Windows, Mac OS X, Linux, FreeBSD

Programming language: Python

Other requirements:

• Python modules – wxPython, pyTables, networkx, numpy, scipy, matplotlib (optional), pycluster or BioPython (optional)

• VTK with python bindings (optional)

• HDF5

License: GNU General Public License version

Any restrictions to use by non-academics: None

## Authors' contributions

JF co-wrote the manuscript and developed most of the front-end Python code, TBK conceived the initial project and wrote the prototypes for kernel density estimation and 3D visualization, CC conceived the project in its current form, designed the architectural framework, developed the backend C++ code and some of the front-end Python, and also co-wrote the manuscript.
